# Unraveling the Mechanisms Linking Physical Activity to Cognitive Function in Older Adults: A Systematic Review of Mediators and Moderators

**DOI:** 10.1192/j.eurpsy.2026.11594

**Published:** 2026-07-31

**Authors:** K. Baliotis, Z. Konstanti, K. Tsamakis, N. Zagorianakou, E. Dragioti, F. Tatsis, T. Bakara Nikou, S. Mantzoukas, M. Gouva, M. Kourakos

**Affiliations:** 1 Scientific Laboratory of Psychology and Person-Centered Care, Department of Nursing, School of Health Sciences, University of Ioannina, Ioannina; 2 Department of Psychiatry, University of Thessaly, Larissa; 3 Research Laboratory of Integrated Health, Care and Well-being, Department of Nursing, University of Ioannina, Ioannina, Greece

## Abstract

**Introduction:**

Physical activity has been consistently associated with improved cognitive functioning in older adults. However, the underlying biological and psychosocial mechanisms through which these benefits are realized remain only partially understood. Previous studies were limited by methodological shortcomings or an overreliance on experimental designs, thus constraining the generalizability of their findings to broader populations.

**Objectives:**

This systematic review aimed to critically evaluate and synthesize the existing literature on the mediating and moderating mechanisms that explain the relationship between physical activity and cognitive performance in adults aged 60 years and older.

**Methods:**

A comprehensive search of PubMed and Scopus databases was conducted from their inception through April 2025. Eligible studies quantitatively assessed physical activity, reported cognitive outcomes, and included analyses of mediation and/or moderation effects. Study designs encompassed randomized controlled trials, longitudinal, cross-sectional, and experimental approaches. Data extraction was performed independently by two reviewers, and methodological quality was assessed based on seven established criteria.

**Results:**

Fifty-six studies met inclusion criteria: 44 examined mediation effects, 10 explored moderation, and 2 addressed both. Strong evidence identified depression and sleep quality as robust mediators of the PA–cognition relationship. In contrast, support for neurocognitive and physiological pathways ranged from limited to moderate. Moderating factors such as genetic predispositions (e.g., APOE genotype), age, comorbidities, and psychosocial context demonstrated inconsistent or weak evidence. Notably, 93% of the included studies were rated as high quality.

**Conclusions:**

Physical activity influences cognitive functioning in older adults through multiple, interrelated mechanisms. Among these, depression and sleep quality emerged as particularly consistent mediators. Future research should prioritize integrative and longitudinal methodologies to unravel causal pathways and guide the development of targeted interventions aimed at preserving cognitive health in aging populations.

**Disclosure of Interest:**

None Declared

